# Fetal 4D flow MRI of the great thoracic vessels at 3 Tesla using Doppler-ultrasound gating: a feasibility study

**DOI:** 10.1007/s00330-022-09167-7

**Published:** 2022-10-22

**Authors:** J. Knapp, M. Tavares de Sousa, A. Lenz, J. Herrmann, S. Zhang, F. Kording, B. Hergert, G. Adam, P. Bannas, B. P. Schoennagel

**Affiliations:** 1 Department of Diagnostic and Interventional Radiology and Nuclear Medicine, University Medical Center Hamburg-Eppendorf, Martinistrasse 52, 20251 Hamburg, Germany; 2 Department of Obstetrics and Fetal Medicine, University Medical Center Hamburg-Eppendorf, Martinistrasse 52, 20251 Hamburg, Germany; 3 Section of Pediatric Radiology, Department of Diagnostic and Interventional Radiology and Nuclear Medicine, University Medical Center Hamburg-Eppendorf, Martinistrasse 52, 20251 Hamburg, Germany; 4 Philips Healthcare, Röntgenstrasse 24, 22335 Hamburg, Germany; 5 Northh Medical GmbH, Röntgenstrasse 24, 22335 Hamburg, Germany

**Keywords:** Fetal MRI, 4D flow, Great thoracic vessels, Doppler-ultrasound gating, Fetal cardiovascular imaging

## Abstract

**Objectives:**

To evaluate the feasibility of Doppler-ultrasound (DUS)-gated 4D flow MRI of the fetal great thoracic vessels at 3T in a clinical setting.

**Methods:**

Sixteen consecutive fetuses (range 30^+4^–38^+5^ weeks) with (*n* = 11) and without (*n* = 5) cardiovascular anomalies underwent 4D flow MRI of the great thoracic vessels at 3T. Direct fetal cardiac gating was obtained using a MR-compatible DUS device. 4D flow MRI–based visualisation and quantification of four target regions (ascending aorta (AAo), descending aorta (DAo), main pulmonary artery (MPA), and ductus arteriosus (DA)) were performed using dedicated software.

**Results:**

Fetal 4D flow MRI of the great thoracic vessels was successful in 12/16 fetuses (75%) by adopting clinical 4D flow MR protocols in combination with direct fetal cardiac DUS-gating. Four datasets were excluded due to artefacts by fetal movement or maternal breathing. 4D flow MRI–derived time-velocity curves revealed typical arterial blood flow patterns in the aorta. 4D flow quantification was achieved for the pre-defined target regions. Average velocity and flow volume were 21.1 ± 5.2 cm/s and 6.0 ± 3.1 mL/s in the AAo, 24.3 ± 6.7 cm/s and 8.4 ± 3.7 mL/s in the DAo, 21.9 ± 6.4 cm/s and 7.8 ± 4.2 mL/s in the MPA, and 23.4 ± 4.7 cm/s and 5.9 ± 3.6 mL/s in the DA, respectively.

**Conclusions:**

Combination of DUS-gating of the fetal heart and 4D flow MRI allows comprehensive visualisation and quantification of haemodynamics in the fetal great thoracic vessels. DUS-gated fetal 4D flow MRI may provide a new diagnostic approach for prenatal assessment of blood flow haemodynamics.

**Key Points:**

• *Fetal cardiac Doppler-ultrasound (DUS) gating and 4D flow MRI can be successfully combined*.

• *DUS-gated fetal 4D flow MRI allowed visualisation and evaluation of streamline directionality, illustration of blood flow variations, and pulsatile arterial waveforms in the target vessels*.

• *4D flow MRI–based visualisation and quantification of the fetal great thoracic vessels were successful and flow metrics agreed with echocardiographic reference values*.

**Supplementary Information:**

The online version contains supplementary material available at 10.1007/s00330-022-09167-7.

## Introduction

Dynamic fetal cardiovascular magnetic resonance imaging (MRI) with high spatio-temporal resolution has been successfully applied using an external MR-compatible Doppler-ultrasound (DUS) device for fetal cardiac gating [1–3]. Until now, 4D flow MRI is established for visualisation and quantification of complex flow haemodynamics only in the postnatal period [4–6]. The combination of direct fetal cardiac gating and 4D flow MRI for the visualisation and quantification of haemodynamics in the human fetus at 3 Tesla has not been investigated so far.

Until recently, fetal cardiovascular MRI was limited by major technical challenges. The main reason was the lack of a gating signal of the fetal heart that is necessary to allow for dynamic imaging with high spatio-temporal resolution. A solution was the introduction of an external MR-compatible DUS device that records the fetal heartbeat and generates a gating signal for synchronisation of the cardiac cycle with MR data acquisition (similar to electrocardiogram gating in adults) [1]. DUS-gating was applied for both anatomical and functional imaging of the fetal cardiovascular system [2, 3, 7].

Phase-contrast (PC) MRI is the reference standard for the quantification of blood flow haemodynamics in the postnatal period [8–10]. 4D flow MRI allows co-registration of morphologic images with flow data and provides time-resolved sets of 3D volumes over a whole cardiac cycle [4, 11]. 4D flow MRI provides additive flow information (e.g. visualisation of flow alterations) and allows comprehensive flow analysis in any vessel or cardiac plane of the acquisition volume. In the fetus, 4D flow MRI could be clinically advantageous over echocardiography as flow acquisition is independent from the fetal intrauterine position.

The aim of this study was to investigate the feasibility of fetal 4D flow MRI in a clinical setting by combining fetal cardiac DUS-gating with 4D flow MRI at 3T for visualisation and quantification of flow haemodynamics in the fetal great thoracic vessels.

## Material and methods

### Study population

This prospective study was approved for pregnant women having fetuses with or without congenital cardiovascular diseases by the local research ethics committee, and all participants gave written informed consent prior to examination.

Sixteen pregnant women with singleton fetuses were asked to participate in the study and to undergo fetal cardiovascular MRI including a 4D flow MRI acquisition. Eleven fetuses had pre- and/or postnatal echocardiographic diagnosis of congenital cardiovascular diseases and two had central nervous system pathology. Three pregnant women without any known fetal anomaly were enrolled. Average gestational age was 35^+0^ weeks (range 30^+4^–38^+5^ weeks), and average maternal age was 33.8 years (range 30–42 years).

### Fetal cardiovascular MRI

Fetal cardiovascular MRI was performed on a clinical 3T system (Ingenia, Philips) using a 32-channel phased array torso coil placed on the maternal abdomen. Pregnant women were positioned in left lateral or supine position according to individual preference. Axial and coronal T2-weighted images of the fetus were acquired during free maternal breathing to identify fetal thoracic anatomy (TE = 80 ms; TR = 2431 ms; FOV = 350 × 301 mm^2^; acquired spatial resolution = 1.2 × 1.4 × 4.0 mm^3^; acquired matrix size = 292 × 216 (reconstructed = 432 × 432); slices = 15; sensitivity encoding (SENSE) factor = 2; number of signal average (NSA) = 1; scan duration = 36 s).

Direct fetal cardiac gating was performed using a MR-compatible DUS-sensor (smart-sync, northh medical GmbH). The recorded DUS-signal of the fetal heart was transferred to the MR unit and served as a gating signal [1].

DUS-gated non-contrast 4D flow MR data were acquired over the entire fetal cardiac cycle in parasagittal orientation (parallel to the aortic arch) with full volumetric coverage of the fetal great thoracic vessels [12, 13]. While the basic Cartesian imaging sequence was kept the same, slight variations of the scan protocols, particularly in spatial and temporal resolutions, were included for feasibility tests. Typical scan parameters were as follows: TE = 1.8–2.0 ms; TR = 2.9–4.0 ms; FOV = 300 × 300 × 25–35 mm^3^; acquired spatial resolution = 1.8 × 1.8 × 2 mm^3^ to 2.5 × 2.5 × 2.5 mm^3^ (reconstructed = 1.0 × 1.0 × 1.0 mm^3^ to 1.25 × 1.25 × 1.25 mm^3^); number of slices = 20–28 (depending on individual fetal anatomy); flip angle = 5°; NSA = 1–2; velocity encoding (VENC) in all three dimensions = 120–200 cm/s; acquired temporal resolution = 26–80 ms (interpolated to 10–56 cardiac phases) [4, 14]. A vendor implementation of the compressed sensing technology, termed compressed SENSE [15], in the standard scanner software, was used with a factor of 4 to 6 for scan time reduction. Average scan time of fetal 4D flow MRI was 2:33 ± 1:25 min. The pregnant women were advised for shallow breathing to minimise motion artefacts; no respiratory gating or additional motion compensation techniques were used.

In three fetuses, additional 2D flow MRI measurements were acquired (TE = 2.9 ms, TR = 4.6 ms, FOV = 280 × 230 mm, FA = 10°, slice thickness = 5 mm, acquired voxel size = 2.5 × 2.5 × 5 mm (reconstructed voxel size = 1.17 × 1.17 × 5 mm), cardiac phases = 35, velocity encoding factor = 120 cm/s, NSA = 1). Dependent on fetal heart rates, temporal resolution was 12–13 ms and scan duration was 12 s.

Comparison of 4D and 2D flow parameters in the ascending aorta (AAo), descending aorta (DAo), and main pulmonary artery (MPA) of three fetuses was assessed. Bland-Altman analysis was performed for stroke volume, mean flow, mean velocity, and peak velocity.

### 4D flow MRI data analysis

4D flow data were corrected for Maxwell terms, eddy currents, and phase aliasing in accordance with current consensus recommendations [4]. 4D flow MRI datasets were automatically reconstructed online at the scanner at the end of the examination with the vendor-provided software. The reconstruction time of each 4D flow MRI dataset was 2–3 min. In a next step, a dedicated 3D visualisation software (GTFlow 3.2, GyroTools LLC) was used offline to render 3D phase-contrast MR angiograms of the fetal great thoracic vessels for each dataset. Rendering, 3D visualisation, and data analysis took on average 30 min.

For quantitative 4D flow analysis, time-velocity curves were generated and average and peak velocities and flow volumes were quantified by manually placing analysis planes at four defined target regions: (1) ascending aorta (AAo) at the level of the main pulmonary artery (MPA), (2) descending thoracic aorta (DAo) at the level of the MPA, (3) MPA, and (4) ductus arteriosus (DA) that acts as a physiological right-left shunt between MPA and DAo. Velocities and flow volumes in the AAo, DAo, MPA, and DA were expressed as averages from all datasets. The maximum transverse diameters of the DAo were measured in the axial or coronal T2-weighted images and averages from all datasets were calculated.

## Results

Fetal 4D flow MRI allowed for visualisation of all four target regions AAo, DAo, MPA, and DA in 12/16 cases (75%). Three datasets were inadequate for further analysis due to severe fetal motion during data acquisition and thus inadequate DUS-gating. Another dataset was excluded from analysis due to severe maternal breathing artefacts (Table 1).

**Table 1:** Overview of study population

Fetus	Gestational age (weeks)	Fetal pathology	4D flow MRI analysis possible
1	38^+1^	Ventricular septal defect	Yes
2	30^+4^	None	Yes
3	31^+3^	Aortic coarctation	No
4	33^+4^	Bicuspid aortic valve with aortic stenosis	No
5	37^+4^	None	Yes
6	31^+2^	Central nervous system anomaly	Yes
7	33^+3^	Right-sided aortic arch	Yes
8	33^+4^	Hypoplastic aortic arch	No
9	38^+3^	Narrow aortic isthmus	Yes
10	32^+5^	Bicuspid aortic valve with aortic stenosis	Yes
11	35^+6^	Tetralogy of Fallot	Yes
12	37^+1^	Atrial septal defect	Yes
13	28^+3^	Ventricular septal defect (suspected)	No
14	38^+5^	Hypoplastic aortic arch (suspected)	Yes
15	35^+0^	None	Yes
16	33^+5^	Central nervous system anomaly	Yes

Repositioning of the DUS-sensor on the maternal abdomen with scan repeat due to gross fetal movement was required in three cases (19%). In these three cases, the acquired images were adequate for analysis.

### Fetal 4D flow MRI — Visualisation

**Fig. 1:**
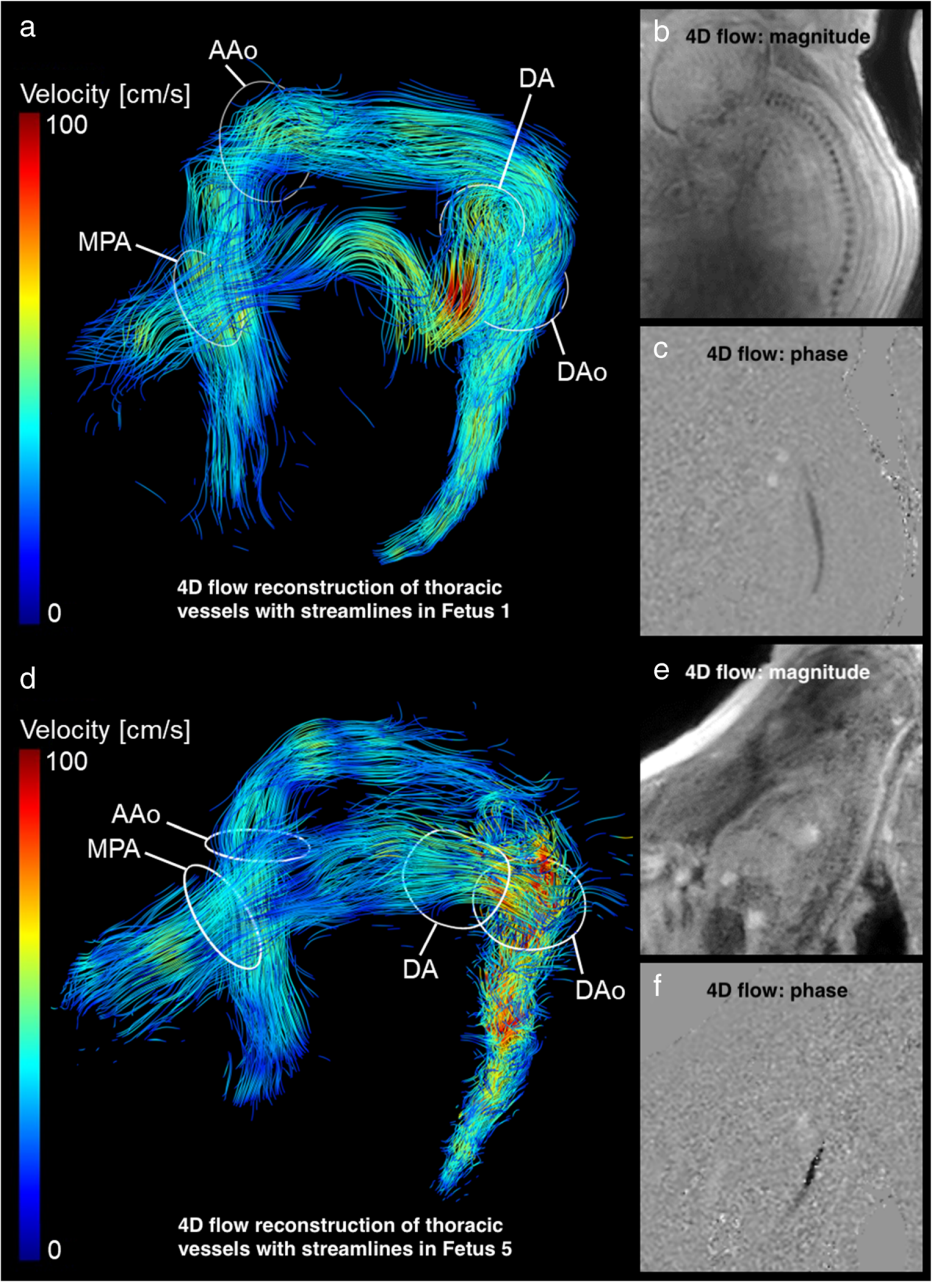
4D flow MRI–based characterisation of flow dynamics (end-diastolic phase) of the great thoracic vessels in two fetuses of similar gestational age employing different spatial resolutions. In fetus 1 (**a**–**c**) (gestational age 38^+1^ weeks), the acquired spatial resolution was 2.5 × 2.5 × 2.5 mm^3^ and in fetus 5 (**d**–**f**) (37^+4^ weeks), the acquired spatial resolution was 1.8 × 1.8 × 2.5 mm^3^. Magnitude (B + E) and phase (C + F) images of the fetal aorta in sagittal view illustrate few motion artefacts in fetus 1 (B + C). 4D flow MRI–based reconstruction of the great thoracic vessels in right antero-lateral view (A + D) allows adequate and similar delineation of fetal thoracic vessels with both spatial resolutions. Velocity-coded streamlines indicate blood flow direction and velocity. The white rings indicate analysis planes for quantitative measurements in the four target regions ascending aorta (AAo), descending aorta (DAo), main pulmonary artery (MPA), and ductus arteriosus (DA). See also Supplementary Material (movies of A and D). Note: Fetus 1 (**a**–**c**) was postnatally diagnosed with a ventricular septal defect. This region, however, is not displayed in the image

Fetal great thoracic vessels were successfully visualised in 12/16 (75%) fetuses by 4D flow MRI–based velocity-coded streamlines indicating blood flow direction and velocity at different time points throughout the cardiac cycle (Fig. 1 and Supplementary Material (movie)).

**Fig. 2:**
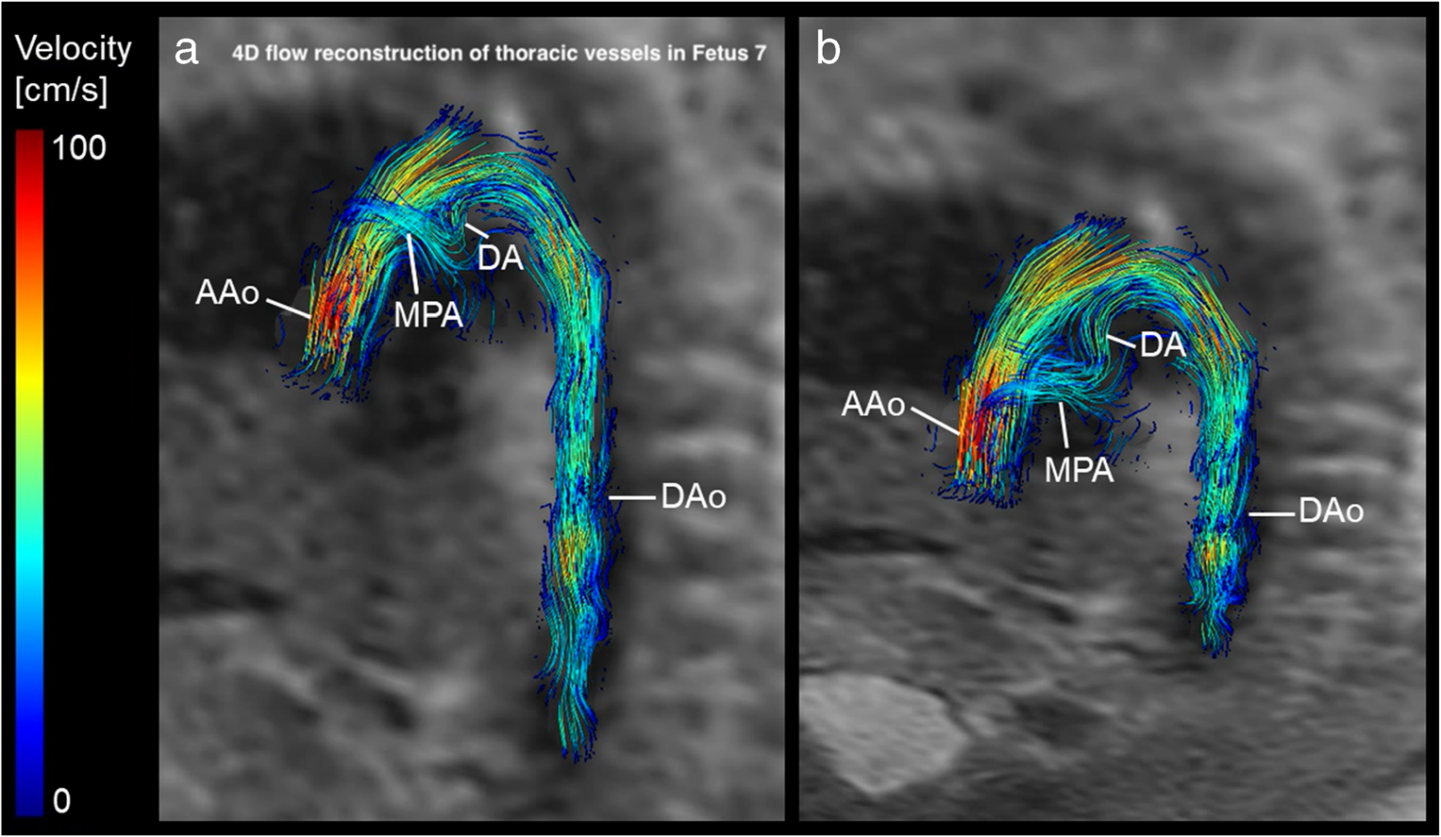
4D flow MRI–based reconstruction with T2-weighted image overlay (systolic phase) in sagittal (**a**) and left cranio-lateral view (**b**) of the fetal great thoracic vessels with known echocardiographic diagnosis of right-sided aortic arch (gestational age 33^+3^ weeks). 4D flow MRI–based reconstruction with various angulation of the thoracic vessel anatomy showed right-sided aortic arch (in combination with transversal T2-weighted imaging) and ductus arteriosus (DA) originating from the main pulmonary artery (MPA) (**b**). DUS-gated 4D flow MRI indicated no double aortic arch anomaly. AAo, ascending aorta; DAo, descending aorta

One fetus (gestational age 33^+3^) had prenatal echocardiographic diagnosis of right-sided aortic arch; however, prenatal echocardiography could not exclude double aortic arch anomaly. 4D flow MRI visualisation of the great thoracic vessels did not indicate double aortic arch anomaly (Fig. 2).

### Fetal 4D flow MRI — Quantification

**Fig. 3:**
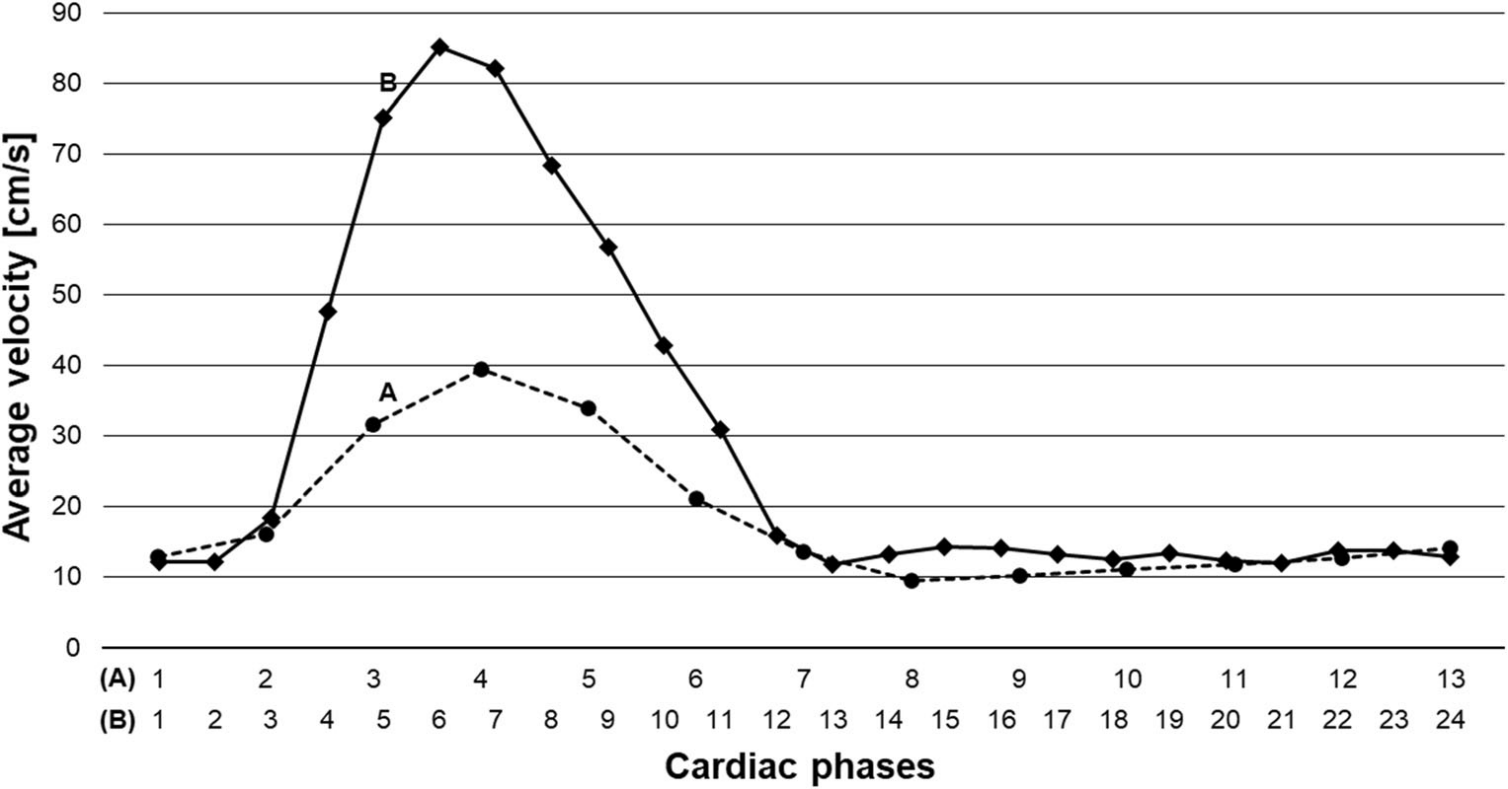
Example of time-velocity curves of the ascending aorta in a healthy fetus (A, dashed line) and a fetus with atrial septal defect (B, black line) but without any vascular pathology. Both fetuses had similar gestational age (37^+4^ and 37^+1^ weeks), heart rate (138 and 135 bpm), and cardiac cycle length (435 and 444 ms). The acquired temporal resolution was higher for fetus B (27 ms, interpolated to 24 cardiac phases) compared to fetus A (50 ms, interpolated to 13 cardiac phases) according to the modified scan protocols. Systolic peaks were lower for fetus A, potentially reflecting the lower temporal resolution and thus lower sensitivity to detect the systolic peak velocity

4D flow MRI provided datasets in 12/16 (75%) fetuses for flow quantification in all four target regions AAo, DAo, MPA, and DA. Time-velocity curves revealed typical arterial blood flow patterns with strong early systolic peaks and low positive diastolic blood velocities in the aorta (Fig. 3).

Mean and peak velocities and flow volumes in each of the four target regions are summarised in Table 2. Peak velocities in the DAo ranged from 30.4 to 75.8 cm/s. Maximum transverse diameter of the DAo was 8.4 ± 1.5 mm.

**Table 2:** 4D flow quantification in the fetal great thoracic vessels

	AAo	DAo	MPA	DA
Mean velocity (cm/s)	21.1 ± 5.2 (14.9–29.8)	24.3 ± 6.7 (15.8–38.8)	21.9 ± 6.7 (12.9–33.3)	23.4 ± 4.7 (15.3–32.4)
Peak velocity (cm/s)	46.6 ± 14.8 (25.0–85.1)	48.7 ± 17.5 (30.4–75.8)	42.4 ± 12.3 (22.3–62.8)	46.6 ± 12.2 (27.0–67.8)
Mean flow volume (mL/s)	6.0 ± 3.1 (2.7–12.2)	8.4 ± 3.7 (3.3–14.5)	7.8 ± 4.2 (0.8–17.7)	5.9 ± 3.6 (1.7–13.8)
Peak flow volume (mL/s)	21.6 ± 12.0 (9.1–44.8)	22.2 ± 9.5 (7.6–35.6)	21.7 ± 12.0 (4.0–52.4)	17.8 ± 11.0 (4.0–42.2)

*AAo*, ascending aorta; *DAo*, descending aorta; *MPA*, main pulmonary artery; *DA*, ductus arteriosus. Mean values ± standard deviations and ranges are provided

Bland-Altman analysis of 4D and 2D flow parameters revealed a relative bias for stroke volumes and mean flows of 7.0% (± 95% limits of agreement: −34%/48%) and 6.9% (± 95% limits of agreement: −39%/53%), respectively. For peak and mean velocities, significantly worse results were achieved (Table 3).

**Table 3:** Comparative 4D and 2D flow MRI–derived parameters in three fetuses

	*Mean velocity (cm/s)*		*Stroke vol. (mL)*		*Mean flow (mL/s)*		*Peak velocity (cm/s)*	
	4D	2D	4D	2D	4D	2D	4D	2D
DAo (fetus 14)	19.6	15.5	5.3	4.5	11.6	10.2	85.9	74.8
DAo (fetus 15)	16.1	17.8	3.7	3.5	8.4	8.6	56.9	85.9
DAo (fetus 16)	15.8	17.2	2.2	3.3	4.2	7.9	60.1	96.9
AAo (fetus 14)	16.0	5.8	1.9	1.8	4.2	3.8	90.9	55.8
AAo (fetus 15)	14.9	11.0	2.9	3.4	6.6	8.1	63.3	55.9
AAo (fetus 16)	17.3	11.2	1.7	2.5	5.3	5.9	93.9	71.5
MPA (fetus 14)	26.5	18.3	8.1	7.6	17.7	17.2	82.2	103.7
MPA (fetus 15)	14.2	9.3	3.3	3.5	7.6	8.3	61.2	53.1
MPA (fetus 16)	19.2	10.5	2.7	2.5	6.6	5.7	56.6	58.1
*Bias*	−34.2%		7.0%		6.9%		−0.2%	
*95%-LLoA*	−97.0%		−33.9%		−39.2%		−62.1%	
*95%-ULoA*	28.5%		47.9%		53.0%		61.6%	

*DAo*, descending aorta; *AAo*, ascending aorta; *MPA*, main pulmonary artery; *vol.*, volume. Bland-Altman analysis was performed

**Fig. 4:**
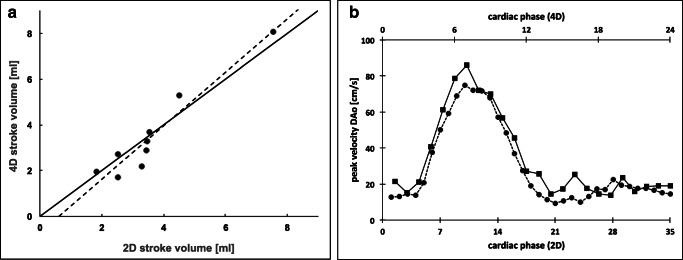
**a** Comparison of 4D and 2D stroke volumes of the DAo, AAo, and MPA in three fetuses revealed high correlation (*r* = 0.96, *p* < 0.001). **b** Representative 4D and 2D peak velocities over the cardiac cycle are illustrated for the DAo in one fetus (fetus 14), revealing good agreement in this example. Additional *x*-axis is provided due to the different number of cardiac phases for 4D and 2D flow sequences (35 vs. 24 phases). DAo, descending aorta; AAo, ascending aorta; MPA, main pulmonary artery

The results from the Bland-Altman plots were confirmed by a highly significant correlation between 4D and 2D stroke volumes (*r* = 0.96, *p* < 0.001) (Fig. 4a). Representative 4D and 2D peak flow velocities over the cardiac cycle are illustrated (Fig. 4b).

## Discussion

Our feasibility study demonstrated successful combination of direct fetal cardiac DUS-gating and 4D flow MRI for visualisation and quantification of blood flow haemodynamics in the fetal great thoracic vessels. Visualisation and correct directionality of streamlines in the target vessels were obtained in all fetuses with successful 4D flow MRI acquisitions. Furthermore, reflection of blood flow variations between the four target vessels and the observed pulsatile arterial waveforms confirm proof of concept of this fetal 4D flow MRI framework. Intra-individual comparison of 4D and 2D flow MRI–derived quantitative parameters in three fetuses demonstrated principal association of quantitative metrics. However, validation and the clinical benefit cannot be claimed by this feasibility approach.

Direct DUS-gating of the fetal heart offers the ability to adjust for intra-individual fetal heart rate variations during the acquisition and provides immediate image review after MR data acquisition. In some cases, however, the DUS-signal of the fetal heart may be lost due to gross fetal movement, which was the reason for incomplete 4D flow datasets in three fetuses in this study.

Quantification of fetal 4D flow MRI datasets revealed time-velocity curves with typical arterial blood flow patterns in the aorta. Quantitative measurements of average and peak velocity and flow volume in the four target vessels were successfully performed. 4D flow MRI measurements were in agreement with echocardiographic reference ranges in the second half of pregnancy, e.g. in the DAo with average velocities of 15.8–38.8 cm/s vs. 20–47.5 cm/s and peak velocities of 30.4–85.9 cm/s vs. 48.2–110.3 cm/s, and in the MPA with average velocities of 12.9–33.3 cm/s vs. 10.8–19.9 cm/s and peak velocities of 22.3–62.8 cm/s vs. 36.0–63.3 cm/s [16, 17]. Especially the assessed peak velocities reflected lower ranges of echocardiographic reference values. Notably, underestimation of peak velocities by PC-MRI of up to 30% is a well-known phenomenon [18–22]. Lower temporal resolutions of MRI compared to echocardiography may contribute to this phenomenon, as a lower temporal resolution is less sensitive to accurately detect velocity peaks.

Comparison of 4D and 2D flow MRI–derived parameters was performed in three target vessels (AAo, DAo, and MPA) of three fetuses. The results from the Bland-Altman analysis or the Pearson correlation are very promising that fetal 4D stroke volumes and mean flows reflect reliable results. Interpretation of the comparative 4D and 2D measurements is limited as only three fetuses were examined. Future studies in larger populations are needed to confirm these preliminary analyses of quantitative flow parameters.

4D flow MRI average velocity and flow volume in the DAo were also in accordance with reported 2D PC-MRI flow measurements at 1.5T (15.8–38.8 cm/s vs. 8.6–36.8 cm/s and 3.3–14.5 mL/s vs. 3.2–14.6 mL/s) [2]. In a different study, 2D PC-MRI demonstrated highest average flow volume for the MPA, followed by the DAo, AAo, and DA [23]. In our study, average flow volume was slightly higher in the DAo than in the MPA, followed by the AAo and DA. From our intra-individual comparisons and comparison with recent literature, we assume that metrics of fetal 4D flow quantification appear reasonable in the context of this feasibility study.

Technical considerations and sequence parameters play an important role in optimising a 4D flow MR sequence protocol for fetal application. Based on these initial results, imaging parameters of fetal 4D flow MRI will be discussed in the following. In this feasibility approach, the intention was to apply clinically available 4D flow MRI sequences in combination with fetal cardiac DUS-gating. Parameters of 4D flow MRI sequences were modified so that reasonable parameter combinations were applied throughout the study. Parameters were adapted according to the small dimensions of the fetal cardiovascular system and high heart rates around 140 bpm (e.g. temporal resolutions ranged from 26 to 80 ms). Of course, lower temporal resolutions appear less appropriate to fully resolve the reported fetal cardiac cycle lengths between 330 and 545 ms [24], baring the risk to miss the actual velocity peaks and with resulting underestimation of velocity measurements. This relation could contribute to the finding of a lower systolic arterial peak in a fetus with lower temporal resolution (Fig. 3). For dynamic MR imaging of the fetal cardiovascular system, temporal resolutions of 12–83 ms have been applied, whereas “high” temporal resolutions were considered to be < 50 ms [25]. From our experience, we would suggest temporal resolutions of 20–30 ms for fetal 4D flow MRI. With respect to spatial resolution, we think that voxel sizes between 2.0 and 2.5 mm are well suited for fetal 4D flow MRI as voxel size is recommended not to exceed one-third of the whole vessel diameter [26]. These voxel sizes reflect the compromise to account for the small fetal dimensions but to preserve adequate signal-to-noise ratio. Choice of VENC values in this study (120–200 cm/s) were not informed by prior echocardiography. We consider a VENC value of 120 cm/s appropriate for fetal imaging based on peak velocities provided by literature [16].

The implementation of compressed sensing at a 3T scanner allowed us to establish an acquisition scheme tailored to 4D flow MRI in the fetus that revealed good image quality in considerably short acquisition times with an average of 2:33 min. Current technical developments, i.e. motion compensation, may contribute to increase diagnostic quality and accuracy of fetal flow quantifications [27].

A potential limitation of our study is that we did not apply a single 4D flow MR sequence protocol. However, the aim of this study was to combine cardiac DUS-gating with 4D flow MRI in the fetus by optimising clinically available 4D flow MR sequences that are ready to use. The comparison of quantitative 4D and 2D flow MRI–derived parameters was performed in only three fetuses. However, validation of quantitative parameters was not the aim of this feasibility study.

In conclusion, this study provided proof of concept for the combination of fetal cardiac DUS-gating and 4D flow MRI. This innovative method has the potential to provide diagnostic information of blood flow haemodynamics and vascular connections in the fetal great thoracic vessels. Further validation of 4D flow MRI–derived quantitative flow parameters is required in larger fetal populations.

## Supplementary Information

ESM 1 (MP4 5042 kb)

ESM 2 (MP4 5998 kb)

## Abbreviations

2D, 3D, 4D: Two-three-four-dimensional
AAo: Ascending aorta
DA: Ductus arteriosus
DAo: Descending aorta
DUS: Doppler-ultrasound
FOV: Field of view
MPA: Main pulmonary artery
MR(I): Magnetic resonance (imaging)
NSA: Number of signal average
PC: Phase-contrast
SENSE: (Compressed sensing) sensitivity encoding
TE: Echo time
TR: Repetition time
VENC: Velocity encoding

## Declarations

### Guarantor

The scientific guarantor of this publication is Bjoern Schoennagel.

### Statistics and biometry

No complex statistical methods were necessary for this paper.

### Study subjects or cohorts overlap

Some of the study subjects have been previously reported at a conference.

Zhang S, Knapp J, Cronenberg R et al (2021) Clinical fetal cardiovascular MRI based on Doppler ultrasound gating at 3T and 1.5T: on a technical aspect of imaging pulse sequence optimization. *Proc Intl Soc Mag Reson Med* 2021 29:0349

Knapp J, Kording F, Tavares de Sousa M et al (2021) Fetale 4D-Fluss-MRT mittels Doppler-Ultraschall Gating bei 3 Tesla. Deutscher Roentgenkongress der Deutschen Roentgengesellschaft e.V. 2021 102:17

### Methodology

• prospective

• experimental

• performed at one institution

## References

[1] Kording F, Yamamura J, de Sousa MT (2018). Dynamic fetal cardiovascular magnetic resonance imaging using Doppler ultrasound gating. J Cardiovasc Magn Reson.

[2] Schoennagel BP, Yamamura J, Kording F (2019). Fetal dynamic phase-contrast MR angiography using ultrasound gating and comparison with Doppler ultrasound measurements. Eur Radiol.

[3] Tavares de Sousa M, Hecher K, Yamamura J (2019). Dynamic fetal cardiac magnetic resonance imaging in four-chamber view using Doppler ultrasound gating in normal fetal heart and in congenital heart disease: comparison with fetal echocardiography. Ultrasound Obstet Gynecol.

[4] Dyverfeldt P, Bissell M, Barker AJ (2015). 4D flow cardiovascular magnetic resonance consensus statement. J Cardiovasc Magn Reson.

[5] Lenz A, Petersen J, Riedel C (2020). 4D flow cardiovascular magnetic resonance for monitoring of aortic valve repair in bicuspid aortic valve disease. J Cardiovasc Magn Reson.

[6] Riedel C, Lenz A, Fischer L (2021). Abdominal applications of 4D flow MRI. Rofo.

[7] Tavares de Sousa M, Hecher K, Kording F (2021). Fetal dynamic magnetic resonance imaging using Doppler ultrasound gating for the assessment of the aortic isthmus: a feasibility study. Acta Obstet Gynecol Scand.

[8] Markl M, Frydrychowicz A, Kozerke S, Hope M, Wieben O (2012). 4D flow MRI. J Magn Reson Imaging.

[9] Stankovic Z, Allen BD, Garcia J, Jarvis KB, Markl M (2014). 4D flow imaging with MRI. Cardiovasc Diagn Ther.

[10] Azarine A, Garcon P, Stansal A (2019). Four-dimensional flow MRI: principles and cardiovascular applications. Radiographics.

[11] Weinrich JM, Lenz A, Girdauskas E, Adam G, von Kodolitsch Y, Bannas P (2020). Current and emerging imaging techniques in patients with genetic aortic syndromes. Rofo.

[12] Kording F, Schoennagel B, Ruprecht C, Tavares de Sousa M, Yamamura J, Giese D (2019). Fetal cardiac 4D phase-contrast MRI using Doppler ultrasound gating to visualize fetal hemodynamics in utero: preliminary results. Proc Intl Soc Mag Reson Med.

[13] Zhang S, Knapp J, Cronenberg R et al (2021) Clinical fetal cardiovascular MRI based on Doppler ultrasound gating at 3T and 1.5T: on a technical aspect of imaging pulse sequence optimization. Proc Intl Soc Mag Reson Med 2021 29:0349

[14] Neuhaus E, Weiss K, Bastkowski R, Koopmann J, Maintz D, Giese D (2019). Accelerated aortic 4D flow cardiovascular magnetic resonance using compressed sensing: applicability, validation and clinical integration. J Cardiovasc Magn Reson.

[15] Meister RL, Groth M, Jurgens JHW, Zhang S, Buhk JH, Herrmann J (2022) Compressed SENSE in pediatric brain tumor MR imaging : assessment of image quality, examination time and energy release. Clin Neuroradiol. 10.1007/s00062-021-01112-3 10.1007/s00062-021-01112-3 PMC9424145 34994810

[16] Bahlmann F, Wellek S, Reinhardt I, Krummenauer F, Merz E, Welter C (2001). Reference values of fetal aortic flow velocity waveforms and associated intra-observer reliability in normal pregnancies. Ultrasound Obstet Gynecol.

[17] Fittschen M, Reinhard I, Wellek S, Friedrichs S, Bahlmann F (2014). Advanced dynamic Doppler flow of the pulmonary artery in a normal population: reference values from 18 to 41 weeks of gestation calculated by automatic Doppler waveform analysis. Arch Gynecol Obstet.

[18] Baledent O, Fin L, Khuoy L (2006). Brain hydrodynamics study by phase-contrast magnetic resonance imaging and transcranial color doppler. J Magn Reson Imaging.

[19] Engvall J, Sjoqvist L, Nylander E, Thuomas KA, Wranne B (1995). Biplane transoesophageal echocardiography, transthoracic Doppler, and magnetic resonance imaging in the assessment of coarctation of the aorta. Eur Heart J.

[20] Seitz J, Strotzer M, Wild T (2001). Quantification of blood flow in the carotid arteries: comparison of Doppler ultrasound and three different phase-contrast magnetic resonance imaging sequences. Invest Radiol.

[21] Shibata M, Sakuma H, Isaka N, Takeda K, Higgins CB, Nakano T (1999). Assessment of coronary flow reserve with fast cine phase contrast magnetic resonance imaging: comparison with measurement by Doppler guide wire. J Magn Reson Imaging.

[22] Stadlbauer A, van der Riet W, Globits S, Crelier G, Salomonowitz E (2009). Accelerated phase-contrast MR imaging: comparison of k-t BLAST with SENSE and Doppler ultrasound for velocity and flow measurements in the aorta. J Magn Reson Imaging.

[23] Prsa M, Sun L, van Amerom J (2014). Reference ranges of blood flow in the major vessels of the normal human fetal circulation at term by phase-contrast magnetic resonance imaging. Circ Cardiovasc Imaging.

[24] Jansz MS, Seed M, van Amerom JF (2010). Metric optimized gating for fetal cardiac MRI. Magn Reson Med.

[25] Roy CW, Seed M, Macgowan CK (2017). Accelerated MRI of the fetal heart using compressed sensing and metric optimized gating. Magn Reson Med.

[26] Lotz J, Meier C, Leppert A, Galanski M (2002). Cardiovascular flow measurement with phase-contrast MR imaging: basic facts and implementation. Radiographics.

[27] Goolaub DS, Xu J, Schrauben E (2021). Fetal flow quantification in great vessels using motion-corrected radial phase contrast MRI: comparison with Cartesian. J Magn Reson Imaging.

